# Post-Acute Sequelae of COVID-19: The Potential Role of Exercise Therapy in Treating Patients and Athletes Returning to Play

**DOI:** 10.3390/jcm12010288

**Published:** 2022-12-30

**Authors:** Luna Cavigli, Chiara Fusi, Marta Focardi, Giulia Elena Mandoli, Maria Concetta Pastore, Matteo Cameli, Serafina Valente, Alessandro Zorzi, Marco Bonifazi, Antonello D’Andrea, Flavio D’Ascenzi

**Affiliations:** 1 Department of Medical Biotechnologies, Division of Cardiology, University of Siena, 53100 Siena, Italy; 2 Department of Cardiac, Thoracic, Vascular and Public Health Sciences, University of Padova, 35128 Padova, Italy; 3 Department of Medicine, Surgery and Neuroscience, University of Siena, 53100 Siena, Italy; 4 Department of Cardiology and Intensive Coronary Care, Umberto I Hospital, 84014 Nocera Inferiore, Italy

**Keywords:** COVID-19, SARS-CoV-2 infection, PASC, exercise intolerance, exercise prescription, rehabilitation programs, athletes

## Abstract

Post-acute sequelae of coronavirus disease 19 (COVID-19) (PASC) describe a wide range of symptoms and signs involving multiple organ systems occurring after severe acute respiratory syndrome coronavirus 2 (SARS-CoV-2) infection, representing a growing health problem also in the world of sport and the athletic population. Patients with PASC have new, returning, or persisting symptoms four or more weeks after the infection. Among the most frequent symptoms, patients complain of fatigue, dyspnea, exercise intolerance, and reduced functional capacity that interfere with everyday life activity. The role of exercise programs in PASC patients will be identified, and upcoming studies will establish the magnitude of their benefits. However, the benefits of exercise to counteract these symptoms are well known, and an improvement in cardiopulmonary fitness, functional status, deconditioning, and quality of life can be obtained in these patients, as demonstrated in similar settings. Based on this background, this review aims to summarise the current evidence about the PASC syndrome and the benefit of exercise in these patients and to provide a practical guide for the exercise prescription in PASC patients to help them to resume their functional status, exercise tolerance, prior activity levels, and quality of life, also considering the athletic population and their return to play and sports competitions.

## 1. Introduction

Severe acute respiratory syndrome coronavirus 2 (SARS-CoV-2) infection rapidly spread and impacted worldwide. Post-acute sequelae of coronavirus disease 19 (COVID-19) (PASC) describe a wide range of health problems occurring after acute infection [1,2,3]. Patients with PASC have new, returning, or persisting symptoms four or more weeks after the infection [2]. Timepoint definition for the post-acute period varies among different guidelines between at least 4 weeks and 3 months after the infection [4,5]. Generally, the term Long COVID Syndrome (LCS) is used to describe signs and symptoms that continue or develop over time after acute COVID-19 infection and may take many months to resolve [6,7]. The reported prevalence of LCS differs among studies and countries [3,8,9]. However, a higher prevalence has been observed in hospitalised patients [8,9] compared with community reports [10], reflecting the role of the severity of acute illness, the presence of comorbidities, and persistent symptoms [3,8,9,10].

PASC is characterised by a wide range of symptoms and signs involving multiple organ systems, including the respiratory, neurological, cardiovascular, gastrointestinal, dermatological, endocrine, genitourinary, and musculoskeletal systems [3]. Among the most frequently reported symptoms in PASC, patients complain of fatigue, dyspnea, and exercise intolerance that interfere with everyday life or worsen after even minor physical or mental efforts [11,12]. Multiple mechanisms for PASC have been proposed, but the pathophysiology underlying the syndrome is not fully understood, and more data are needed to help clinicians in the diagnosis, treatment, and prognosis [2]. Moreover, clinical investigations may not identify the correspondence between reported symptoms and specific organ dysfunction or may identify abnormalities not related to the symptoms experienced by the patients [2], implying the need for further research and a multidisciplinary approach [2,3]. PASC patients complain of a loss of freedom to engage with routine activities, resulting in an early onset of fatigue; they have expectations of how the healthcare system could support them with physical activity, but questions about how to manage exercise have challenged the expertise of medical professionals [13].

Hence, the knowledge on COVID-19 issues is still limited, and many studies are under development. In this review, we performed a literature search to analyse the studies related to the effects of exercise training and rehabilitation programs on post-COVID-19 complications in order to report the principles of personalised exercise prescription in patients suffering from this syndrome, also considering the athletic population and their return to play and sports competitions.

The current review article was based on a literature search of electronic databases (mainly PubMed). The search terms were designed based on the main following keywords: COVID-19; SARS-CoV-2 infection; PASC; LCS; exercise intolerance; fatigue; deconditioning; exercise prescription; rehabilitation programs; exercise rehabilitation; cardiorespiratory fitness; resistance training; strength training; athletes.

## 2. The Benefit of Exercise on PASC Patients

PASC and its common clinical manifestations, such as fatigue and exercise intolerance, could be explained at least in part by deconditioning and reduced exercise capacity [14,15,16]. In a post-COVID-19 study, patients with reduced exercise capacity demonstrated a lower peak of aerobic capacity (VO_2max_ < 85% predicted) at cardiopulmonary exercise test (CPET), early aerobic threshold, lower levels of performance, and earlier termination of the test as compared to patients with normal exercise capacity, whereas pulmonary function tests and parameters of ventilatory efficiency or gas exchange were normal, suggesting a high degree of deconditioning [17]. Pathophysiology of deconditioning in PASC patients might be related to the direct effects of viral load on muscle tissue and prolonged rest [17]. Postural orthostatic tachycardia syndrome (POTS), characterised by orthostatic intolerance and an exaggerated increase in heart rate (HR) upon standing, has been described in PASC patients, although much remains to be understood about the relationship between PASC and POTS [2]. Prolonged supine position as in bed rest may lead to orthostatic intolerance with multiple possible mechanisms, such as fluid redistribution (central fluid shift and neurohumoral-mediated reduction in blood and plasma volume), changes in loading condition, and reduced preload leading to a negative cardiovascular remodelling [18]. This adverse remodelling associated with deconditioning, relative to reduced blood volume, cardiac output, and increased heart rate, can be counteracted by physical activity, which in turn increases blood volume, cardiac size, and mass. Therefore, exercise training may potentially have beneficial effects on the recovery of PASC patients, and preliminary studies support the positive impact of exercise rehabilitation on cardiorespiratory fitness [6,17,19,20,21,22]. Moreover, it seems necessary for early intervention during the ongoing symptomatic COVID-19 phase to prevent PASC syndrome and avoid the chronicity of fatigue [23,24,25]. Hence, COVID-19 patients can benefit from an early rehabilitation program after hospital discharge, composed of resistance and aerobic exercises, as it may improve their functional capacity and quality of life (QoL) (reducing stress and mental disorders) [26,27,28,29]. Daynes et al. documented in post-COVID-19 patients the feasibility and efficacy in terms of improved clinical outcomes of walking capacity and symptoms of fatigue, cognition, and respiratory symptoms of a 6-week rehabilitation program comprehensive of aerobic exercise (walking/treadmill-based), strength training of upper and lower limbs, and educational discussions with the progression of the exercise component in line with patient’s symptoms [20]. Barbara et al. recently demonstrated the benefit of aerobic and resistance training (8 weeks) on cardiorespiratory and musculoskeletal fitness in LCS patients evaluated after 3 months of hospital discharge [30].

A multidisciplinary rehabilitation program, including both physical training and psychological treatment, was conducted by a multidisciplinary team including physical trainers, nurses, psychologists, cardiologists, and sport medicine physicians in PASC patients (symptoms continuing after 4 or more weeks after the end of the infection) [6]. The training program included three sessions per week of 90 min duration, with endurance training (continuous moderate-intensity training corresponding to 60–80% of VO_2peak_ obtained during CPET), followed by resistance strength training, conducted at a variable load of 30–50% of the 1-RM. After the multidisciplinary rehabilitation program, COVID-19 residual symptoms significantly decreased, and significant improvements in upper and lower limb muscular strength, cardiopulmonary parameters, perceived physical and mental health, depression, and anxiety were observed [6].

Telerehabilitation has been proposed as a good alternative when a face-to-face program is not possible. Patients showing post-COVID-19 fatigue have benefited from a telerehabilitation program at home, including aerobic and strength exercises, leading to benefits on persistent symptoms, such as fatigue, and on several parameters of physical capacities such as VO_2max_, maximal aerobic power, or walking distance [23].

Post-COVID-19 patients may experience altered respiratory function and impaired diffusion capacity lasting for months after the infection [22]. Hence, pulmonary rehabilitation, proven to reduce dyspnea and increase exercise capacity and QoL in patients with chronic obstructive pulmonary disease or interstitial lung diseases, has been applied in patients post-COVID-19 [22,31]. Patients with various degrees of lung impairments after COVID-19 and with comorbidities, including mild-to-moderate heart disease, hypertension, diabetes, obesity, lung disease, and osteoporosis, undergoing pulmonary rehabilitation through respiratory muscle training (breathing control and thoracic expansion) and with or without endurance training, demonstrated an increase of exercise capacity, dyspnea, and QoL, in the absence of adverse events [22,32,33,34]. In a prospective observational cohort study on PASC outpatients undergoing a personalised pulmonary rehabilitation program, consisting of exercise training sessions split up into endurance, strength, and inspiratory muscle training over 6 weeks, three times per week for 3–4 h each, improvements in exercise capacity, functional status, dyspnea, fatigue, and quality of life were observed [21].

Functional consequences of acute SARS-CoV-2 infection, particularly physical impairment, highlight the importance of rehabilitation in acute, post-acute, and chronic phases of infection to relieve symptoms, improve the quality of life, and get back to previous physical fitness [35]. Notably, maximal exercise capacity is independently and inversely associated with the likelihood of hospitalisation due to COVID-19, and physical inactivity is associated with a higher risk for severe COVID-19 outcomes [36,37].

In a safe and effective rehabilitation program, physical activity should be prescribed with a tailored approach and personalised based on individual characteristics.

Moreover, the positive effects of rehabilitation in deconditioned patients, consequently to prolonged bedrest or reduction of the usual physical activities, both in-hospital and early after discharge, are well-documented by studies on cardiac rehabilitation after cardiac events [38] and on exercise training programs in patients recovering from other severe acute respiratory syndromes [39,40]. Indeed, previous studies demonstrated the effectiveness of an exercise training program on the cardiorespiratory and musculoskeletal performance of patients who were recovering from severe acute respiratory syndrome (SARS) [39,40]. Furthermore, rehabilitation programs can improve indices of left ventricular (LV) diastolic and systolic dysfunction, LV global longitudinal strain (GLS), left atrial (LA) strain at echocardiography in cardiac diseases such as chronic heart failure, hypertension, myocardial infarction, and coronary artery disease [41,42,43].

Therefore, patients with cardiovascular complications after COVID-19 could benefit from rehabilitation programs, as observed in other cardiac clinical contexts.

## 3. How to Prescribe Exercise in PASC Patients: The FITT Approach

The benefits of physical activity are well known, improving cardiovascular and non-cardiovascular profiles, quality of life, and reducing mortality [44,45]. Exercise prescription should be tailored according to the patient’s characteristics, the drugs administered, the personal history, the response to exercise, the aims to reach, and the different health profiles to improve [46,47]. Hence, the patient should be evaluated in his/her complexity to adapt an exercise program appropriate to its characteristics, health problems, symptoms, and consequences of COVID-19. Moreover, the aim and outcomes should be clearly identified before prescribing exercise. Indeed, for example, in obese patients, the objective is not only the body-weight loss but also improving other critical health-related aspects (e.g., blood pressure, blood lipid profile, insulin sensitivity, systemic inflammatory markers, physical fitness, and quality of life) [47]. Hence, in PASC patients, exercise prescription should aim to improve functional capacity and cardiopulmonary and psychological symptoms (i.e., fatigue, dyspnoea, exercise intolerance, and anxiety). A multidisciplinary team should establish a general treatment plan adapted for each patient according to the clinical presentation.

In this context, based on the studies related to exercise training in COVID-19 patients and exercise prescription in other conditions [38,45,46], we suggest some general principles of exercise prescription in adult subjects suffering from PASC syndrome and without contraindications to physical activity (i.e., orthopaedic limitations, severe cardiovascular contraindications to exercise training, psychiatric or neurological disorders, etc.) [6].

The exercise prescription is based on the so-called ‘FITT’ model (frequency, intensity, time, and type) [48].

Frequency is the number of sessions/weeks; patients can start with two sessions/week until reaching a target of 3–5 times/week [48]. In PASC patients, a training program including three sessions per week led to cardiopulmonary benefits without adverse events [6,21].

Intensity is the amount of energy expenditure/time unit during training sessions. Different methods can be used to define the intensity of exercise and can be divided into subjective and objective methods. The formers include the Borg scale (perceived exertion scale) and the talk test (the degree of ability to talk during exercise) [48,49]. The objective methods include the percentage of maximal HR or HR reserve (HRR, i.e., the difference between HRmax and resting HR) identified by exercise testing and the percentage of VO_2max_ [45,50]. The CPET gives the unique opportunity to delineate the ventilatory thresholds (VTs), the first (VT_1_) and second (VT_2_) ventilatory thresholds, which represent the most reliable method to identify the correct intensity of aerobic exercise [50,51]. The aerobic and anaerobic thresholds can also be obtained by lactate testing (determination of lactate levels in the blood via withdrawal from the ear lobe): the first threshold can be identified at approximately 2 mmol/L of blood lactate while the second approximately corresponds to 4 mmol/L [45,52]. This method could be particularly helpful for clinicians who do not own the CPET but only the ergometric test, which associates the lactate test as an alternative method to determine the thresholds and intensity of exercise.

The intensity of aerobic exercise can be classified as light, moderate, high, and very high. Moderate intensity of aerobic exercise is slightly above or around VT_1_; therefore, the HR value corresponding to the VT_1_ can be derived and used as an objective indicator for prescribing moderate aerobic exercise [45,51].

In PASC patients, continuous moderate-intensity training led to significant improvements in cardiopulmonary parameters and symptoms, perceived physical and mental health, depression, and anxiety [6,27,30,53,54]. Moreover, moderate-intensity exercise positively affects the immune system and inflammation; on the contrary, intensive exercise can increase the risk of inflammation and exacerbate virus infection, such as COVID-19 [55,56,57]. Therefore, it is advisable to prefer moderate-intensity exercise, especially at the beginning of the program [19].

The definition of exercise intensity based on derived percentages rather than individually determined VTs may result in an incorrect estimation of exercise intensities that could be potentially dangerous in patients taking beta-blockers [51]. Therefore, the CPET allows for an accurate, individualised exercise prescription and for patients with PASC permits to identify the grade of any functional limitation and understanding whether the causes of functional limitation and symptoms are attributable to cardiac, ventilatory, vascular, pulmonary, or peripheral problems [50]. Moreover, the patients can be re-evaluated by CPET to assess the improvement of functional capacity, symptoms, and the positive effects of exercise, so that it may be a helpful monitoring system for exercise capacity and cardio-ventilatory limitations in subjects admitted to a rehabilitation program [58].

Time represents the duration of a training program in weeks or months, training days/week, training session times/day, and duration of training sessions in hours [48]. In PASC patients, rehabilitation programs included two–three exercise sessions a week, from 20 min to 60 min each, for 4–12 weeks [26,30,53,54].

Type of exercise includes aerobic, resistance training, strengthening, respiratory, flexibility, and balance exercises. Aerobic training can be continuous or interval-based (i.e., short bouts of exercise at high intensities, interspersed with recovery periods) [48]. Endurance training can include running, cycling, swimming, walking, etc. PACS patients with tachycardia, exercise/orthostatic intolerance, and/or deconditioning could be unable to tolerate upright exercise (i.e., power walking, jogging), which may worsen fatigue [2]. Therefore, recumbent or semi-recumbent exercise (e.g., rowing, swimming, or cycling) is preferable in these patients with transition to upright exercise over time as orthostatic intolerance resolves [2].

Resistance training can either be isometric (unchanged muscle length) or isotonic (change in length of the muscle) [48]. It is important to define the intensity of resistance training by defining the repetition maximum (RM), i.e., the maximum weight a person can lift throughout a range of motion. Moderate intensity of resistance training can be defined as 30–50% of 1 RM [45,48]. In the study of Barbara et al., LCS patients demonstrated an increase in musculoskeletal fitness after 8 weeks of resistance training (for the lower extremity: leg extension/flexion, abduction/adduction, and leg press; for the upper extremity: push-up/pull-down; for the core muscles; abdomen, back) [30]. Resistance training prescription load was defined as 40% of 1RM, two sets (three sets for the last two weeks), and 12 repetitions for each muscle group, and the progression of resistance training was confirmed after four weeks by defining the new 1 RM values to be used to continue training [30]. In other studies, exercise intensity varied from 30 to 80% of 1-RM, and the average exercise intensity was between 50 and 70% of 1-RM; the number of repetitions ranged from 8 to 20 repetitions, and the mean repetitions were between 8 and 12 repetitions [26]. In addition, the number of sets in resistance training varied from two to three [26]. Among the different approaches that can be used, the one suitable for the patient must be individualised according to fitness and comorbidities.

Flexibility and balance exercises have been included in the training program in post-COVID-19 patients [27,29,30,53,59]. Flexibility is the range of motion (ROM) of one or several joints. Dynamic stretching refers to achieving, on a repeated gradual transition on any part of the body, a progressive increase in ROM and could be performed at the beginning of the exercise as a warm-up phase, while static stretching refers to the ability to maintain the position at the end of the ROM and can be included in the cool-down phase [29,59,60]. The post-COVID-19 programs included a 5 min warm-up phase and 5 min of stretching [29,30,59]. Balance exercises are important to reduce the risk of falls, particularly in the elderly. It is important to choose the type of exercise (single leg stances, toe walking, heel walking, eye–hand or eye–leg coordination, etc.) to improve balance depending on which system (sensory, cognitive, or musculoskeletal system) needs to be worked on [60]. Static and dynamic balance exercises (walking with obstacles, changing directions, or on unstable surfaces) were included in the exercise programs in post-COVID-19 patients improving their physical performance [27].

The progression of exercise training is another pivotal element of exercise prescription. The exercise dose should be increased gradually and frequently, considering the patient’s adaptation to exercise, age, and clinical characteristics [50]. In post-COVID-19 programs, individuals started with two sessions/week, introducing other sessions when appropriate for the patient, starting from 15–30 min up to 60 min for each session. Aerobic exercises were performed at moderate intensity, and increased to high-intensity or interval training when appropriate [26,27,28,29,30,53,54,61,62]. Guidelines recommend a minimum of 150 min per week of moderate-intensity aerobic training [48]. In PACS patients with tachycardia, exercise/orthostatic intolerance, and/or deconditioning, exercise duration should be short initially, and exercise intensity should be at a submaximal level, increasing gradually as tolerance and functional capacity increase [2]. It has been proposed for these patients to start with daily recumbent exercise for only 5–10 min at a level that allows them to speak in full sentences with gradual increases in exercise [2].

For strength activities, the PASC patient should start gradually with one to three sets of 8–10 repetitions, increasing the volume/intensity every week or after four weeks if indicated, defining the new 1RM values to be used to continue training [26,30].

Respiratory rehabilitation is another crucial point in the post-COVID-19 rehabilitation program. Inspiratory muscle training may enhance respiratory muscle strength, improve aerobic capacity, and diminish dyspnea [33,63]. Post-COVID-19 patients demonstrated an improvement in respiratory function after 6-week respiratory rehabilitation training, which included respiratory muscle training (using a hand-held resistance device, three sets with 10 breaths in each set; parameters were set at 60% of the individual’s maximal expiratory mouth pressure), cough exercises (three sets of 10 active coughs), diaphragmatic training (diaphragmatic contractions in the supine position, placing a medium weight (1–3 kg) on the anterior abdominal wall to resist diaphragmatic descent), stretching exercises (the respiratory muscles are stretched under the guidance of a rehabilitation therapist), and home exercises (pursed-lip breathing and coughing training) [32].

Psychological treatment must be tailored to patients’ specific symptoms and based on cognitive behavioural therapy. In addition, patients can be taught about individual relaxation techniques, such as muscular relaxation, breath control, and imaginative relaxation, particularly in patients showing anxiety and acute stress symptoms [6,64].

Once an exercise program is prescribed, strict compliance must be stressed to verify the correct performance of the training. After a few months, a new evaluation is required to adjust the exercise prescription, reassess intensity and VTs, and assess improvement of functional capacity and symptoms [45,50].

**Figure 1:**
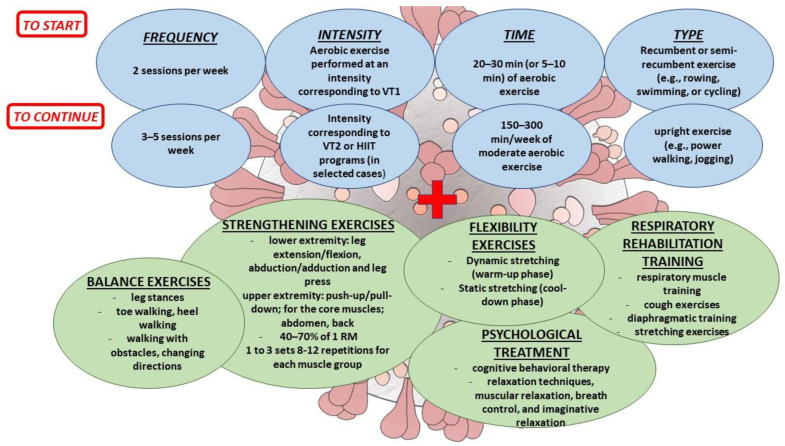
General principles of personalised exercise prescription in PASC patients.

The basic principles of personalised exercise prescription in PASC patients based on the studies available are shown in Figure 1.

## 4. SARS-CoV-2 Infection and Sport

The SARS-CoV-2 infection strongly impacted the world of sport, both for the suspension of competitions and the spread of the infection among athletes [65,66]. Although the athletic population is usually young and healthy, cardiovascular complications have been reported in these subjects after the infection [67,68,69,70,71], particularly during the first wave of the pandemic. Initial data reported a higher complication rate among competitive athletes, with 15% and 39% of athletes demonstrating myocarditis and pericarditis, respectively [67,68]. However, other studies and registries documented a lower prevalence of myocarditis (0.7–1.4%) [72,73,74].

In this context, there is an international consensus on screening athletes with previous SARS-CoV-2 infection before the resumption of sport to exclude proarrhythmic cardiac substrate that may pose a risk to the athlete during exercise [2,71]. Although different modalities are recommended, a general agreement exists on the opportunity to indicate the testing for the return-to-play (RTP) evaluation primarily based on the course of the COVID-19 disease and the presence of cardiopulmonary symptoms [71,75,76,77]. Indeed, the presence of cardiopulmonary symptoms, ECG abnormalities, and arrhythmias found during the RTP screening at rest or during exercise represent red flags that must lead to further investigations to assess the presence of cardiac complications and proarrhythmic cardiac substrate, particularly myocarditis [69,71].

Currently, there is limited research reporting the symptoms of PASC among athletes and the recommendations for athletes returning to competition/training who have experienced long COVID symptoms. A large U.S. registry of college athletes showed that 1.2% were symptomatic beyond 21 days after the infection, while in a smaller U.K. cohort, 14% of young elite athletes experienced symptoms >28 days, and 27% were unable to return to full sports participation at 1 month following initial infection [78,79,80]. Some authors suggest evaluating athletes with PASC with cardiopulmonary symptoms before their RTP with resting ECG, blood tests, echocardiography, adding cardiac magnetic resonance (CMR), CPET, and pulmonary function testing if appropriate [2]. However, these recommendations are not based on solid evidence. Therefore, further research is required to identify the real prevalence of PASC syndrome in the athletic population and to evaluate the best management for athletes suffering from this syndrome [81]. Case series reported athletes suffering from PASC, POTS, and exercise and orthostatic intolerance, demonstrating the utility of performing CPET in making the diagnosis and emphasizing the role of graded exercise training to treat this condition [80]. Hence, athletes with PASC may not readily resume their prior activity levels in a short time, so it seems reasonable to recommend a graded exercise program based on a tailored, individualised approach throughout the recovery process, applying the same principles described for the other patients until the resolution of the condition [2].

## 5. Conclusions

Post-acute sequelae of coronavirus disease 19 (PASC) are characterised by a wide range of symptoms and signs involving multiple organ systems representing a growing health problem. Among the most frequent symptoms, patients complain of fatigue, dyspnea, exercise intolerance, and reduced functional capacity that interfere with everyday life activity. The current review demonstrated that multidisciplinary rehabilitation programs can lead to an improvement in cardiopulmonary and musculoskeletal fitness, functional status, deconditioning, fatigue, and quality of life in post-COVID-19 patients. Upcoming studies will implement the knowledge regarding the modalities and the short/long-term effects of exercise in this setting. To date, these results highlight the importance of multidisciplinary rehabilitation programs, based on individualised and tailored exercise prescriptions, to be added to the continuum of care in PASC patients.

## Data Availability

Data availability statements are not applied given that original data were not presented in this manuscript.
